# Apathetic Thyroid Storm Presenting as New-Onset Dilated Cardiomyopathy and Cardiogenic Shock

**DOI:** 10.7759/cureus.108388

**Published:** 2026-05-06

**Authors:** Pragya Bhandari, Tark Abou-Elmagd, Shreebridhi Pande, Nirajan Kandel, Inmaculada Ncogo Alene

**Affiliations:** 1 Internal Medicine, Cape Fear Valley Medical Center, Fayetteville, USA

**Keywords:** cardiogenic shock, cardiomyopathy, nonischemic cardiomyopathy, thyroid storm, thyrotoxicity

## Abstract

Thyroid storm is an uncommon but life-threatening endocrine emergency associated with high mortality, and its diagnosis remains largely clinical due to the lack of universally reliable criteria to distinguish it from severe thyrotoxicosis; atypical or “apathetic” forms are particularly rare and can delay recognition. A 49-year-old man with no prior medical history presented with a two-week history of progressive anasarca, palpitations, and nonspecific gastrointestinal symptoms in the absence of fever or agitation and was found to have atrial fibrillation with rapid ventricular response and decompensated heart failure. Laboratory studies showed a suppressed thyroid-stimulating hormone with markedly elevated free T3 and T4, while echocardiography revealed global hypokinesis with an ejection fraction of 25-30%. His Burch-Wartofsky score of 60 supported the diagnosis of thyroid storm. Despite progression to cardiogenic shock, he improved with rate control, aggressive diuresis, esmolol, methimazole, and glucocorticoids without requiring inotropic support; subsequent outpatient evaluation confirmed Graves’ disease, and at one year, he remains euthyroid with recovering ventricular function. This case highlights that apathetic thyroid storm may present predominantly with severe cardiomyopathy and shock in the absence of classic hyperadrenergic signs, underscoring the importance of early recognition and prompt targeted therapy for favorable outcomes.

## Introduction

Thyroid storm is a rare endocrine emergency with an estimated incidence of 0.57-0.76 cases per 100,000 persons annually in the United States and mortality approaching 7.4% [1]. Mortality remains substantial despite modern critical care management. Diagnosis is primarily clinical, as no universally accepted biochemical thresholds reliably distinguish thyroid storm from severe thyrotoxicosis [2,3]. Scoring systems such as the Burch-Wartofsky Point Scale (BWPS) and the Japanese Thyroid Association (JTA) criteria have been developed to aid diagnosis, yet both rely heavily on clinical findings [4].

Although classic features include hyperthermia, agitation, tachycardia, and multiorgan dysfunction, a minority of patients, estimated at <1%, may exhibit an atypical or “apathetic” presentation lacking hyperadrenergic signs [3]. Such cases are particularly challenging when cardiac manifestations dominate the clinical picture.

We report a case of apathetic thyroid storm presenting as new-onset dilated cardiomyopathy and cardiogenic shock in a previously healthy middle-aged man.

## Case presentation

A 49-year-old man with no significant past medical history presented with a two-week history of progressive generalized edema, palpitations, and nonspecific gastrointestinal discomfort. He denied fever, tremors, anxiety, or heat intolerance. There was no history of prior thyroid disease or cardiac conditions.

On admission, he was tachycardic with an irregularly irregular rhythm and was found to be in atrial fibrillation with rapid ventricular response. Blood pressure was borderline hypotensive. Physical examination revealed elevated jugular venous pressure, bilateral pulmonary crackles, abdominal distention, and 3+ lower-extremity edema, consistent with decompensated heart failure. No obvious goiter or ophthalmopathy was initially noted.

Laboratory testing demonstrated an undetectable thyroid-stimulating hormone (TSH) and elevated free T3 and free T4 (Table 1). Cardiac biomarkers were mildly elevated without ischemic electrocardiographic changes.

**Table 1:** Laboratory findings demonstrating suppressed TSH with markedly elevated free T3 and free T4 TSH, thyroid-stimulating hormone

Test	At presentation	Reference range
TSH	Undetectable	0.450-5.330 uIU/mL
Free T3	7.77 pg/mL	2.0-4.4 pg/mL
Free T4	4.23 ng/dL	0.61-1.12 ng/dL

**Figure 1:**
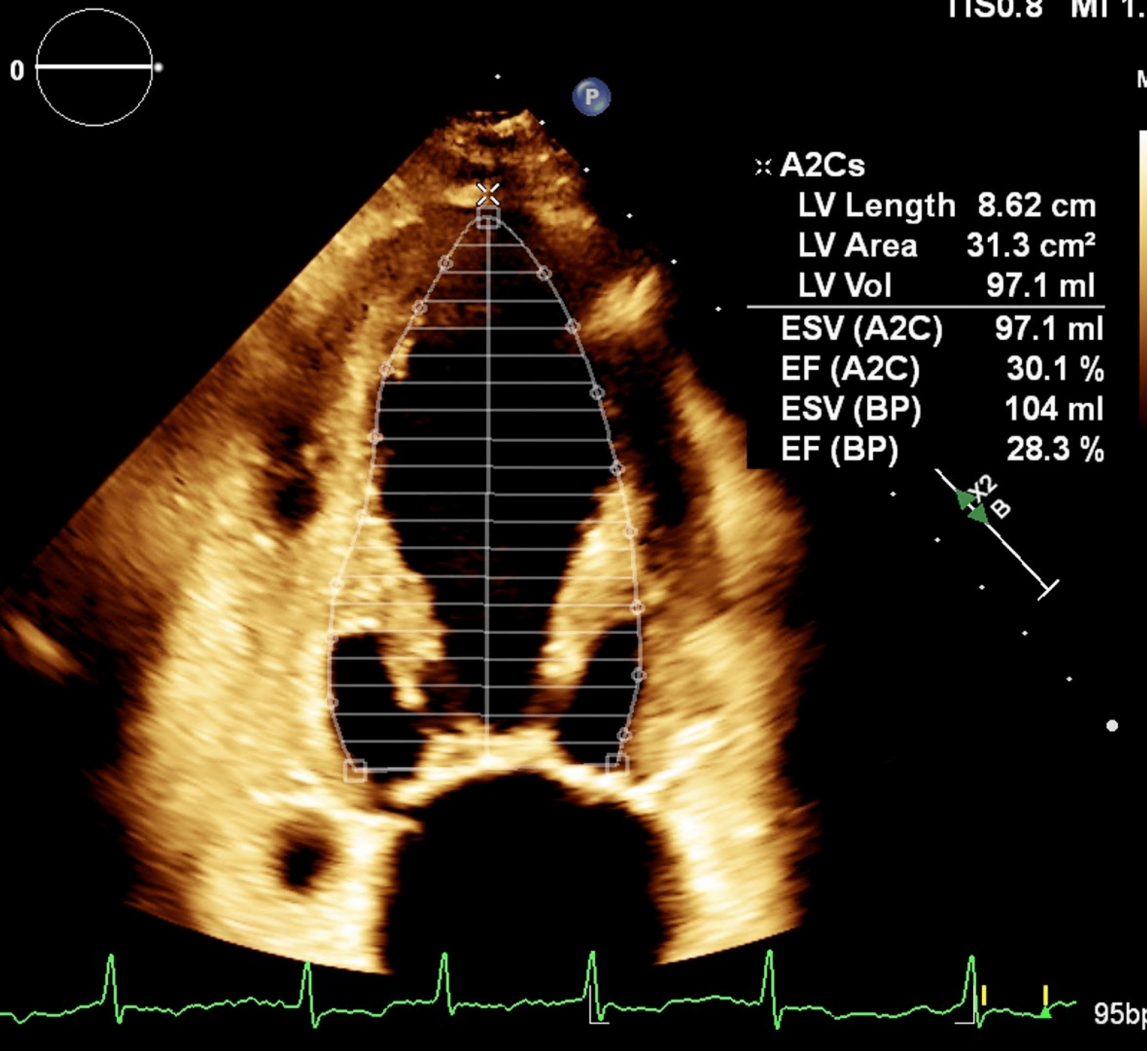
Transthoracic echocardiography showing global hypokinesis with severely reduced LVEF (25-30%), consistent with dilated cardiomyopathy LVEF, left ventricular ejection fraction

**Figure 2:**
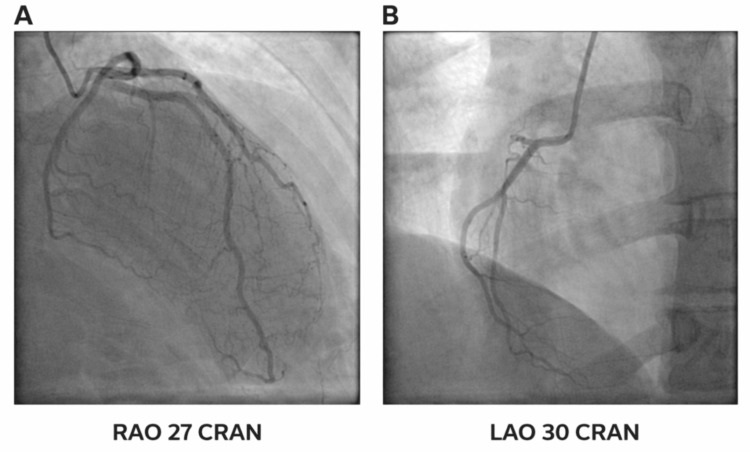
Coronary angiography in RAO cranial view demonstrating a normal left coronary system, including the left anterior descending and left circumflex arteries (A), and LAO cranial view demonstrating a normal right coronary artery (B) LAO, left anterior oblique; RAO, right anterior oblique

Transthoracic echocardiography revealed global hypokinesis with a severely reduced left ventricular ejection fraction (LVEF) of 25-30%, consistent with dilated cardiomyopathy (Figure 1). There were no regional wall motion abnormalities to suggest ischemia. The patient underwent coronary angiography, which ruled out obstructive coronary artery disease as a cause of cardiomyopathy (Figure 2).

Using the BWPS, he scored 60 points, consistent with thyroid storm [4]. During hospitalization, he developed cardiogenic shock, manifested by hypotension and evidence of hypoperfusion. However, he did not require vasopressors or inotropic support.

Management included intravenous esmolol for rate control, aggressive diuresis, methimazole to inhibit thyroid hormone synthesis, and glucocorticoids to reduce peripheral conversion of T4 to T3. Iodine therapy was administered after initiation of antithyroid medication. His heart rate gradually stabilized, volume status improved, and hemodynamics normalized.

Outpatient evaluation confirmed Graves’ disease as the etiology of his thyrotoxicosis. At one-year follow-up, he remains clinically euthyroid with marked improvement in LVEF, consistent with reversible thyrotoxic cardiomyopathy.

## Discussion

Thyroid storm represents the most severe and life-threatening manifestation of thyrotoxicosis and requires immediate recognition and aggressive management. It is characterized by acute decompensation of multiple organ systems due to the profound metabolic and adrenergic effects of excess thyroid hormone. Despite advances in diagnostic tools and treatment strategies, thyroid storm continues to carry significant morbidity and mortality. National inpatient data have demonstrated substantial mortality associated with hospitalizations for thyroid storm, underscoring the importance of early diagnosis and timely therapeutic intervention [1]. Current diagnostic and management recommendations from the American Thyroid Association and the JTA emphasize rapid clinical recognition and prompt initiation of therapy [5,6]. However, unlike many other endocrine emergencies, thyroid storm remains primarily a clinical diagnosis, as no single laboratory test definitively confirms the condition. Clinicians must therefore rely on a combination of biochemical evidence of thyrotoxicosis and characteristic systemic manifestations to guide diagnosis and treatment decisions.

An uncommon but important variant is apathetic thyroid storm, also referred to as atypical thyroid storm. This presentation is rare, occurring in less than 1% of patients with thyrotoxicosis [3]. While it is classically described in elderly individuals, it may also occur in younger patients, making recognition particularly challenging. In contrast to the classic hyperadrenergic presentation characterized by fever, agitation, tremor, and marked tachycardia, patients with apathetic thyroid storm may present with subtle or paradoxical symptoms, including lethargy, weakness, apathy, depression, or unexplained cardiovascular compromise. Several mechanisms have been proposed to explain this atypical presentation. Chronic exposure to high circulating thyroid hormone levels may lead to downregulation of β-adrenergic receptors, reducing the typical hyperadrenergic response. Additionally, prolonged thyrotoxicosis may blunt central nervous system responsiveness and alter metabolic signaling pathways. A predominance of catabolic processes may further contribute to profound fatigue and muscle weakness rather than the hypermetabolic features commonly associated with thyroid storm [6]. Because classic findings such as fever, agitation, and marked sympathetic activation may be absent, the diagnosis is often delayed, which can significantly worsen clinical outcomes.

Thyroid hormones exert profound effects on the cardiovascular system, and cardiac manifestations are among the most clinically significant complications of severe thyrotoxicosis. Excess thyroid hormone increases myocardial contractility and heart rate by enhancing β-adrenergic receptor sensitivity and sympathetic tone. In addition, thyroid hormone decreases systemic vascular resistance through peripheral vasodilation, resulting in increased venous return and elevated cardiac output [5,7]. These hemodynamic changes can lead to a sustained high-output circulatory state, which may initially appear compensatory but can eventually overwhelm myocardial reserve. At the cellular level, thyroid hormone also exerts direct genomic effects on cardiomyocytes, influencing the expression of contractile proteins, calcium-handling channels, and mitochondrial enzymes that regulate myocardial energy utilization [6,8].

In cases of severe or prolonged thyrotoxicosis, these mechanisms may culminate in thyrotoxic cardiomyopathy, a potentially reversible form of dilated cardiomyopathy characterized by ventricular dilation and impaired systolic function [7]. Several pathophysiologic mechanisms have been proposed to explain this phenomenon. Persistent tachycardia may result in tachycardia-induced myocardial stunning, while chronic metabolic stimulation can cause energetic depletion of cardiomyocytes. Mitochondrial dysfunction and oxidative stress may further impair myocardial contractility. Additionally, prolonged high-output physiology can eventually progress to decompensated heart failure with reduced systolic function. Although atrial fibrillation is a well-recognized complication of hyperthyroidism, progression to cardiogenic shock is relatively uncommon and represents a severe manifestation of cardiovascular involvement [8]. In the present case, a combination of rapid ventricular response, sustained high-output physiology, and the direct toxic effects of thyroid hormone on the myocardium likely contributed to the development of severe systolic dysfunction and hemodynamic instability.

Importantly, thyrotoxic cardiomyopathy is often partially or completely reversible once euthyroidism is restored and appropriate cardiovascular support is provided [7]. With effective control of thyroid hormone levels and rate control for associated arrhythmias, many patients experience significant recovery of left ventricular function over time. This reversibility highlights the critical importance of early recognition and targeted therapy, as prompt treatment can significantly improve cardiac outcomes and reduce mortality [9].

This case also illustrates the diagnostic limitations of commonly used scoring systems, including the BWPS and diagnostic criteria proposed by the JTA [4,10]. While these tools are helpful in supporting clinical suspicion, they rely heavily on features such as fever, central nervous system agitation, and overt hyperadrenergic manifestations. In atypical or apathetic presentations, these hallmark findings may be absent, resulting in lower scores and potential underrecognition of thyroid storm. Consequently, clinicians must maintain a high index of suspicion, particularly in patients presenting with unexplained cardiovascular decompensation.

Thyroid storm should therefore be considered in patients presenting with unexplained worsening heart failure, new-onset dilated cardiomyopathy, atrial fibrillation with rapid ventricular response, or cardiogenic shock without an obvious ischemic or structural cause. In such clinical scenarios, prompt assessment of thyroid function is essential. Early identification of thyrotoxicosis can facilitate rapid initiation of antithyroid therapy, beta-blockers, corticosteroids, and supportive measures, which may dramatically improve outcomes. Failure to recognize atypical thyroid storm can delay lifesaving treatment and significantly increase morbidity and mortality.

## Conclusions

Atypical or apathetic thyroid storm is a rare but life-threatening form of thyrotoxicosis that may present without classic hyperadrenergic features, instead manifesting predominantly as severe cardiomyopathy, arrhythmias, or cardiogenic shock, making diagnosis particularly challenging. Because it remains a clinical diagnosis without definitive confirmatory testing, clinicians must maintain a high index of suspicion in patients with unexplained acute heart failure or hemodynamic instability, even in the absence of fever or agitation. This case underscores that thyroid hormone excess can profoundly impair cardiovascular function through both direct myocardial toxicity and sustained high-output physiology; however, this process is often reversible with timely recognition and appropriate therapy. Early initiation of antithyroid treatment, rate control, and supportive measures can lead to substantial recovery of cardiac function and improved survival, highlighting the critical need for prompt evaluation of thyroid function in unexplained cardiovascular collapse and the importance of considering atypical thyroid storm in the differential diagnosis.
